# Breathless on the journey: Unmasking palbociclib-induced pneumonitis in a geriatric breast cancer warrior

**DOI:** 10.5339/qmj.2024.qitc.19

**Published:** 2024-04-01

**Authors:** Abdullah Mohammad Arshad, Mona Babiker, Theeb Sulaiman, Aisha Al Adab

**Affiliations:** 1 Pulmonology Department, Hamad Medical Corporation, Doha, Qatar Email: aarshad1@hamad.qa

**Keywords:** Breast cancer, Palbociclib, Pulmonary, Toxicity, novel, dysnea

## Introduction

Palbociclib is an oral inhibitor of cyclin-dependent kinase 4/6 that has demonstrated efficacy in combination with hormonal therapy for the treatment of hormone receptor-positive metastatic breast cancer. Although palbociclib is generally well tolerated, pulmonary toxicity associated with palbociclib is a rare but potentially serious adverse event.^1,2^

## Case Presentation

A 75-year-old female with stage 4 breast cancer who was on palliative hormonal therapy and palbociclib presented with a one-week history of fever, dry cough, and progressively worsening dyspnea on exertion. No history of hemoptysis, leg swelling, paroxysmal nocturnal dyspnea (PND), or orthopnea was reported. The patient denied sick contacts, recent travel, personal or family history of tuberculosis, or chronic respiratory illnesses. Physical examination revealed bilateral fine crackles, predominantly in the upper lobes, with oxygen requirement at rest.

**Figure 1.**
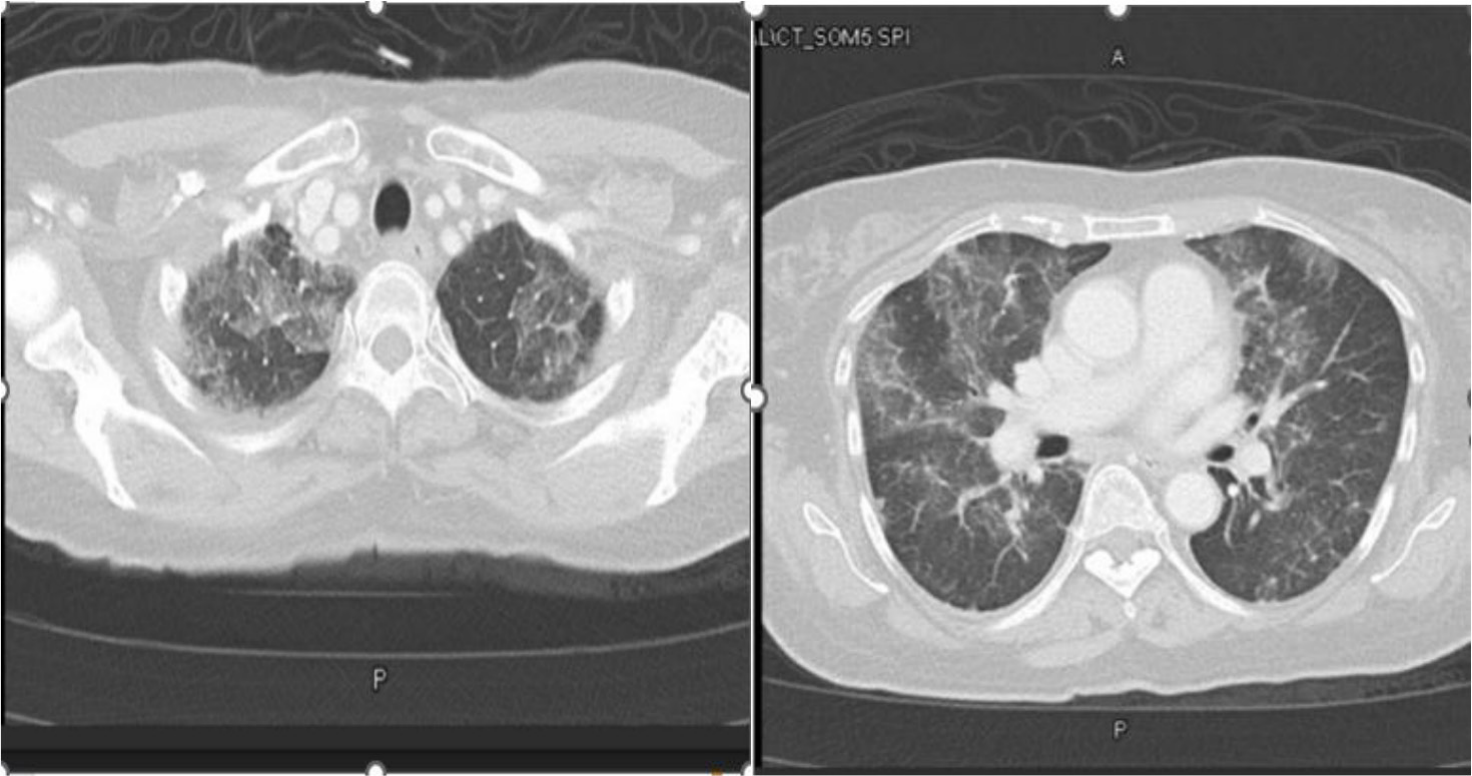
Bilateral patchy ground-glass opacities with some crazy paving patterns.

Laboratory tests showed normal WBC and elevated C-reactive protein (CRP). Chest X-ray showed bilateral opacities, and a high-resolution computed tomography (HRCT) scan revealed ground-glass opacities consistent with pneumonitis (Figure 1).

Bronchoscopy ruled out infectious etiologies. Given the temporal relationship between palbociclib initiation and symptom onset, along with the absence of alternative etiologies, the diagnosis of palbociclib-induced pneumonitis was established. The drug was promptly discontinued, and the patient was started on corticosteroid therapy.

**Figure 2.**
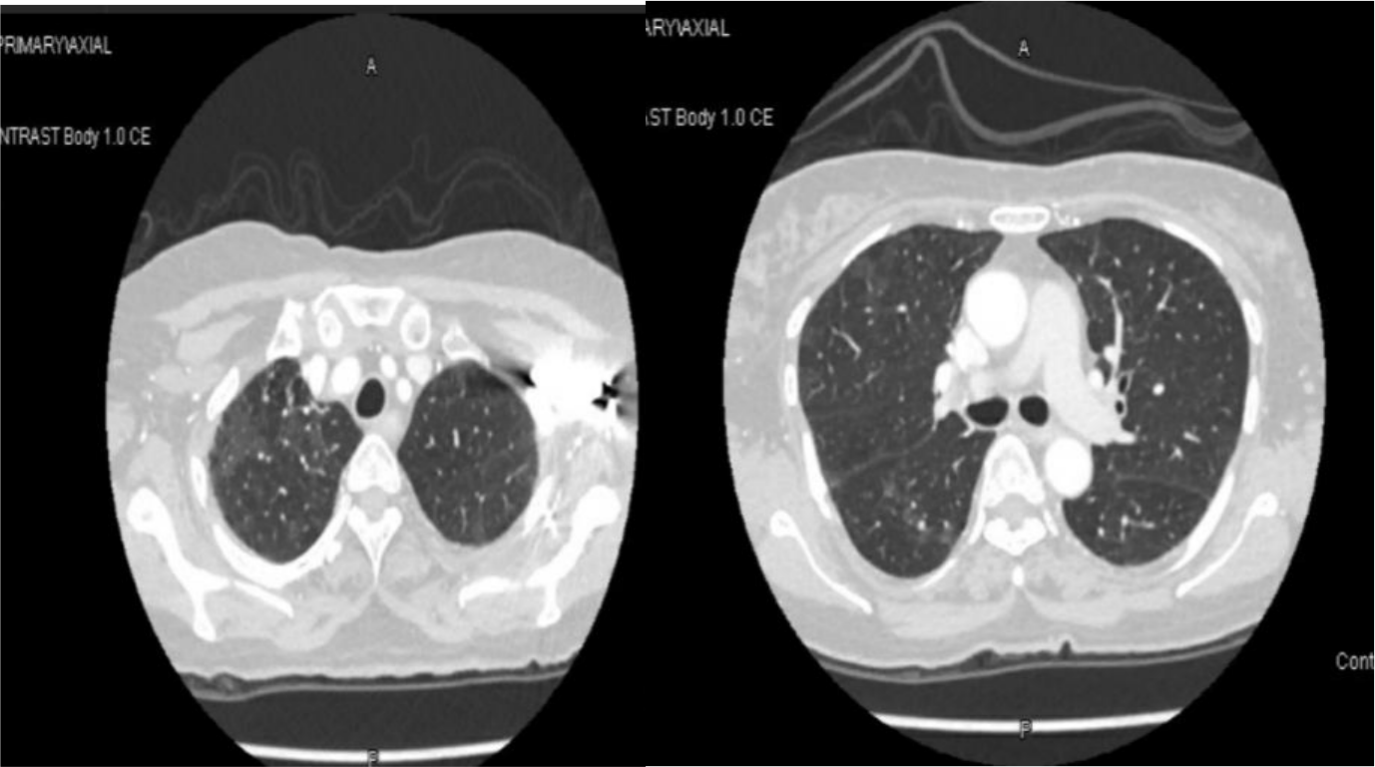
Resolution of previous ground-glass opacities.

The patient showed clinical improvement with resolution of symptoms and radiological findings after discontinuation of palbociclib and initiation of corticosteroids (Figure 2).

## Conclusion

This case highlights the importance of considering drug-induced pneumonitis in cancer patients receiving novel therapies, especially in the context of evolving treatment modalities. Prompt recognition, discontinuation of the offending agent, and appropriate management contribute to favorable outcomes in patients experiencing drug-induced pulmonary toxicity.

## Conflict of Interest

There is no conflict of interest in regards to this abstract.
